# Design and Development of Autotaxin Inhibitors

**DOI:** 10.3390/ph14111203

**Published:** 2021-11-22

**Authors:** Yi Jia, Yan Li, Xu-Dong Xu, Yu Tian, Hai Shang

**Affiliations:** Institute of Medicinal Plant Development, Chinese Academy of Medical Sciences & Peking Union Medical College, Beijing 100193, China; jiayi_91@163.com (Y.J.); w19990921ly@163.com (Y.L.); xdxu@implad.ac.cn (X.-D.X.)

**Keywords:** autotaxin, inhibitor, lysophosphatidic acid, structure design, development

## Abstract

Autotaxin (ATX) is the only enzyme of the ecto-nucleotide pyrophosphatase/phosphodiesterase (ENPP2) family with lysophospholipase D (lysoPLD) activity, which is mainly responsible for the hydrolysis of extracellular lysophosphatidylcholine (LPC) into lysophosphatidic acid (LPA). LPA can induce various responses, such as cell proliferation, migration, and cytokine production, through six G protein-coupled receptors (LPA1-6). This signaling pathway is associated with metabolic and inflammatory disorder, and inhibiting this pathway has a positive effect on the treatment of related diseases, while ATX, as an important role in the production of LPA, has been shown to be associated with the occurrence and metastasis of tumors, fibrosis and cardiovascular diseases. From mimics of ATX natural lipid substrates to the rational design of small molecule inhibitors, ATX inhibitors have made rapid progress in structural diversity and design over the past 20 years, and three drugs, GLPG1690, BBT-877, and BLD-0409, have entered clinical trials. In this paper, we will review the structure of ATX inhibitors from the perspective of the transformation of design ideas, discuss the advantages and disadvantages of each inhibitor type, and put forward prospects for the development of ATX inhibitors in the future.

## 1. Introduction

**Figure 1:**
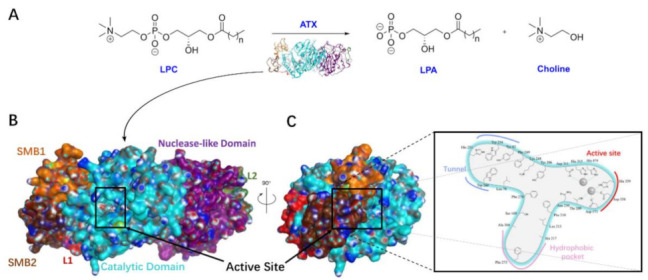
Conversion of LPC into LPA by ATX and overall structure of ATX. (**A**). ATX hydrolyzes lysophosphatidylcholine (LPC) to lysophosphatidic acid (LPA). (**B**). Surface representation of the ATX structure and domains (ID 2XR9). Somatomedin B-like domain 1 (SMB1) in orange; SMB2 in brown; Catalytic Domain in cyan; Nuclease-like Domain in violet; L1 in red; L2 in green. (**C**). The key residues and tripartite interacting site in the T-shaped binding pocket of ATX.

Autotaxin (ATX), one of seven members of the ecto-nucleotide pyrophosphatase/phosphodiesterase family (ENPP2) and the only enzyme of the ENPP family with lysophospholipase D (lysoPLD) activity [1]. It was first isolated from A2058 melanoma cells in 1992 and classified as an autocrine motility factor [2], mainly responsible for the hydrolysis of extracellular lysophosphatidylcholine (LPC) which leads to the production of lysophosphatidic acid (LPA) (Figure 1A).

LPA is a bioactive lipid widely distributed in the body; there are two main pathways for its production, PLA1/2-mediated hydrolysis of phosphatidic acid, and ATX-mediated hydrolysis of LPC, which is the dominant pathway [3,4]. The concentration of LPA in plasma is in the range of 100–164 nM, which is 1/1000 of the LPC’s [5], with a half-life of approximately 3 min; LPA is mainly hydrolyzed by membrane-bound lipid phosphate phosphatases (LPPs) and releases inactive monoacylglycerols [6,7]. Under physiological and pathological conditions, LPA can induce various responses such as cell proliferation, migration, and production of cytokines through six G-protein-coupled receptors (LPA1-6). The LPA signaling pathway is associated with metabolic and inflammatory disorder, and its dysregulation often causes fibrosis [8], liver disease [9], inflammation [10], pruritus [11], metabolic syndrome [12] and cancer [13]. Thus, the development of inhibitors against the ATX-LPA axis is an attractive therapeutic strategy for the treatment of related diseases.

## 2. Structure and Function of ATX

There are a total of five isoforms of ATX already known in mouse and human, which are ATX α, ATX β, ATX γ, ATX δ, and ATX ε [14]. ATX α, isolated from human melanoma cells A2058, contains 915 amino acid residues and was the first subtype to be identified, with less expression in the central nervous system and peripheral tissues [15]. ATX β, 863 amino acid residues long, is the most important subtype, mainly distributed in peripheral tissues and slightly expressed in the central nervous system. In addition to lysoPLD activity, ATX β can also bind to integrins and improve cell proliferation and directional cell motility. These functions are related to the SMB domain, which is mentioned afterwards [16,17,18,19]. ATX δ and ATX ε are the most widespread and stable forms of all ATX subtypes, were originally cloned from ATX β and ATX α, and exist in many organisms, from fish to mammals [20]. In 2011, the crystal structures of ATX in humans and mice were identified [21]. The crystal structures showed that ATX mainly contained two somatomedin B (SMB)-like domains on the same side (N-terminal) (SMB1 and SMB2), nuclease-like domain (NUC) at the other end (C-terminal) and catalytic phosphodiesterase domain (PDE) in the middle; the active site catalyzing the hydrolysis of LPC was located at the center of PDE (Figure 1B). It has been shown that SMB1 and SMB2 are important for catalytic activity, and it is speculated that they are also involved in the subsequent release of LPA [22]. Analysis of the structure of the PDE active site shows that it forms a T-type binding site (Figure 1C), which contains a catalytic site containing two zinc ions and a hydrophobic pocket which is connected to the catalytic site through a hydrophilic shallow groove. The acid head of the substrate of ATX interacts with the two zinc ions, the hydrophilic glycerol part accommodates the shallow groove part and the tail is located on the hydrophobic pocket. On the other side there is a tunnel leading to the PDE [21], which is both hydrophobic and hydrophilic, and can bind steroids [23] and LPA [22,24]. Recent studies have shown that the active ATX can carry LPA to bind on the surface of secreted exosomes [25], and it can be further speculated that ATX bound to LPA binds on the cell surface and subsequently releases LPA to activate LPARs. But molecular dynamics simulations [24] suggest that LPA binding is an independent event in the allosteric tunnel of ATX and does not necessarily mean that the LPA produced by LPC hydrolysis excretes ATX through the tunnel.

## 3. The Potential of ATX as a Drug Target

ATX itself can be a biomarker for many diseases, such as pancreatic cancer [26], Graves’ disease [27], glaucoma [28], acute and chronic liver failure [29], calcific aortic valve stenosis (CAVS) [30], and more. We have mentioned the idea of treating of related diseases by blocking ATX-LPA axis above. Recently, many studies have shown that inhibiting ATX can have a promising therapeutic effect on many diseases, such as the growth and metastasis of various tumors such as oral squamous cell carcinoma (OSCC) [31] and breast cancer [32], peritoneal spread in patients with pancreatic ductal adenocarcinoma (PDAC) [33], cardiovascular disease [34], fibrosis of organs such as kidney, heart and lung [35,36], and central nervous system disease [37,38]. It has been shown that ATX is essential for the development of embryos. ATX may be the only way to produce LPA in the early stage of development [39]. ATX homozygous knockout (KO) embryos die in utero on average at day 9.5 with vascular and neural tube defects [40]. In adult life, ATX is mainly expressed by adipose tissue, reproductive organs and the central nervous system, and a lack of ATX activity leading to lower LPA levels can cause pruritus [41]. Inducible depletion of about 80% of ATX in adult mice does not cause any serious phenotypic defects [42]. It can be assumed that ATX is a rather safe target for various diseases, but it needs more research, as to date there is no information known for humans. This review will summarize ATX inhibitors from the perspective of their developmental history and design ideas.

## 4. ATX Inhibitors Based on Lipid Analogues

Before Hausmann et al. solved the crystal structure of ATX in 2011, the development of ATX inhibitors was limited by the lack of full understanding of the three-dimensional structure of ATX and of the structural diversity of existing ATX inhibitors. There were two main strategies for the development of ATX inhibitors; first, targeting the common active sites of the NPP family known at that time, namely, structures composed of central divalent cations and serine, threonine, or cysteine residues around them [43,44], while the second was mimicking the ATX substrates LPC/LPA or sphingosylphosphorylcholine/sphingosine 1-phosphate (SPC/S1P) based on natural physiological products such as amino acid derivatives, fatty alcohol phosphate (FAP), darmstoff, or sphingosine 1-phosphate (S1P) and so on [45] to produce competitive inhibition.

The former is mainly a series of metal chelators that bind to zinc ions in the active site of ATX, thereby inhibiting its activity, such as EDTA. In 2005, Timothy et al. found that L-histidine (**1**) (compound **1**–**11** in Table 1) at a concentration that is not harmful to cells can inhibit the motility stimulation of ATX on two tumor cell lines from human melanoma (A2058) and human ovarian cancer (SKOV-3). This inhibition can be eliminated by adding excessive zinc ions, which is a non-competitive inhibition. It was the first inhibitor with certain selectivity compared to potent non-specific metal inhibitors; however, the lack of potency and instability in vivo affected its subsequent development [46].

The original lipid-based ATX inhibitors were discovered when researchers looked for LPA receptor inhibitors. Durgam’s group had previously identified FAP (**2**) as the smallest pharmacophore interacting with the LPA receptor [47]. In order to discover LPA receptor agonism and antagonists based on FAP, compound **3** was found to have a complete dose-dependent inhibition of ATX (IC_50_ = 100 nM, LPC) while acting on the LPA receptor at the same time [48]. Compound **4**, obtained by further modification, was still a dual inhibitor of ATX (IC_50_ = 597 nM, LPC) and LPA receptors (LPA3, IC_50_ = 11 nM) [49]. Miller et al. found that the LPA analogue darmstoff (**5**) inhibited ATX (IC_50_ = 97 nM, LPC) while antagonizing LPA3 and activating PPARγ [50]. The subsequent modification of darmstoff revealed that the introduction of a phosphorus atom in the five-membered ring of five could provide better chemical stability while retaining ATX inhibition activity. The final compound **6** (IC_50_ = 220 nM, LPC), inhibited the activity of B16-F10 melanoma cells in mouse lungs [51,52]. In addition, α-bromomethylphosphate (BrP-LPA, **7**) (IC_50_ = 0.7–1.6 μM, LPC) [53], a synthetic analog of S1P (FTY720-P, **8**) (Ki = 0.2 μM, Bis-pNPP) [54], and a tyrosine derivative (**9**) (73% inhibition of ATX at 1 μM) [58], were developed as lipid-like ATX inhibitors. Among all the lipid-like ATX inhibitors, the most potent compound is S32826 (**10**) (IC_50_ = 5.6 nM, LPC) obtained by Ferry et al. during high-throughput screening (HTS) of 13,000 compounds. S32826 had a strong inhibitory effect and selectivity on ATX in vitro. Furthermore, more, plasma ATX activity and the release of LPA had been strongly inhibited by S32826. However, S32826’s kinetic properties and solubility were very poor and could not be applied in vivo. In order to improve the kinetic characteristics of S32826, Jiang [56] et al. and Fisher et al. [55] tried to further modify S32826; however, both failed to successfully perform in vivo experiments. Gupte et al. obtained compound **11** by converting the amide bond into a carbon chain, which decreased the inhibitory potency of ATX (IC_50_ = 170 nM, LPC); however, the metabolism in vivo was highly stable and could effectively inhibit the invasion of MM1 hepatoma cells [57].

From the development of lipid-like ATX inhibitors we can see that most of these inhibitors have the problems with low potency, poor selectivity or poor pharmaceutical properties. Even S32826, with a single nM level of inhibition, is still undruggable due to its solubility and high molecular weight, which fully illustrates the limitations of these inhibitors.

## 5. Small Molecule ATX Inhibitors

### 5.1. First Try

Before the crystal structure of ATX was revealed, most research groups focused on the derivation of natural substrates for the development of ATX inhibitors. In 2008, Saunders et al. performed HTS to find ATX small molecule inhibitors using FS-3 or pNP-TMP as substrates when studying the role of ATX in melanoma cell migration and invasion. They obtained NSC48300 (**12**) (compounds **12**–**19** in Table 2) (Ki = 240 nM) with complete ATX inhibition [59]. During the same period, Parrill’s group used virtual screening and inhibitor-based QSAR for the first time to find small molecule inhibitors, then used FS-3 to determine the in vitro ATX inhibition potency of the preliminarily screened compounds. They obtained lead compounds with different structures, such as xanthene (**13**), phenyldiazenylphenyl (**14**), and isoindole (**15**), which had a potency against ATX greater than 50% at a concentration of 10 μM. Many of these structure fragments, such as dihydropyrimidine and piperazine, can be found in high-potency small molecule ATX inhibitors today. Limited by the diversity of structure, low selectivity, and poor druggability of inhibitors at that time, the QSAR process was not smooth. However, the attempt to develop non-lipid inhibitors was very important to demonstrate the druggability of ATX and was a very positive attempt at that time [60]. In 2010, the same group optimized pipemidic acid compound **16** (IC_50_ = 1.6 μM, FS-3) obtained from virtual screening based on QSAR. They analyzed the selectivity, dose-effect relationship and inhibitory mechanism of and obtained eight compounds with IC_50_ < 10 μM, of which six were competitive inhibitors with Ki = 700 nM–7.1 μM. Compound **17** had the highest potency in the competitive inhibitors (IC_50_ = 900 nM, FS-3, Ki = 0.7 μM). Compound **18** was a non-competitive inhibitor (IC_50_ = 1.5 μM, FS-3), and compound **19** was a mixed inhibitor (IC_50_ = 1.6 μM, FS-3), but this group did not conduct subsequent optimization and in vivo studies [61].

### 5.2. Classic Inhibitor

**Figure 2:**
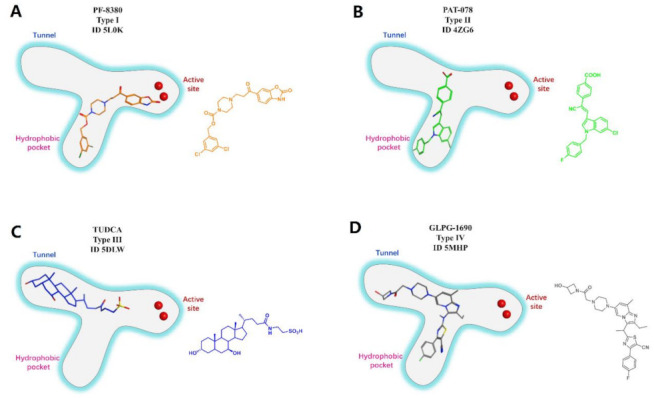
Classification of the reported representative ATX inhibitor types based on binding modes. Left: Schematic diagrams of the binding modes with 3D structures of ATX inhibitor; Right: 2D structures of ATX inhibitor. (**A**). PF-8380, type I, (ID 5L0K); (**B**). PAT-078, type II, (ID 4ZG6); (**C**). TUDCA, type III (ID 5DLW); (**D**). GLPG-1690, type IV, (ID 5MHP).

From 2010 to 2011, Harald et al. developed a series of ATX small molecule inhibitors containing boronic acid head and conducted optimization based on ATX structure, of which the most potent compound HA155 (**20**, compound **20**–**42** in Table 3) (IC_50_ = 5.7 nM, LPC) was the first small molecule ATX inhibitor [62,63,64]. Meanwhile, PF8380 (**21**) (IC_50_ = 1.7 nM, LPC), developed by Pfizer, was the most potent small molecule inhibitor of ATX. With the crystal structure of ATX and the co-crystal structure of small molecule inhibitors such as HA155 and PF8380 in complex with ATX solved [21], it was found that the binding of these two types of compounds to ATX possessed a similar mode; that is, they possessed (a) acidic groups, such as boric acid, carboxylic acid, phosphoric acid or other heterocyclic rings containing nitrogen, oxygen, sulfur, which could bind to zinc ions or amino acid residues such as threonine Thr209 (mATX, Thr210 in hATX) at the catalytic site, (b) a hydrophobic tail which could bind to the lipophilic pocket composed of amino acid residues such as Phe210, Leu213, Ala217, Phe273, and Ala304, and (c) a core spacer guiding the overall molecular orientation. The ATX inhibitors composed of these three parts became the most classic structure, and this binding mode with ATX was called the type I binding mode (Figure 2A). Many research groups optimized and designed other inhibitors according to the binding mode of HA155 and PF8380. Next, this review will summarize the common structures of the three parts in this type of inhibitor.

#### 5.2.1. Head Anchored at the Active Site

**Scheme 1:**
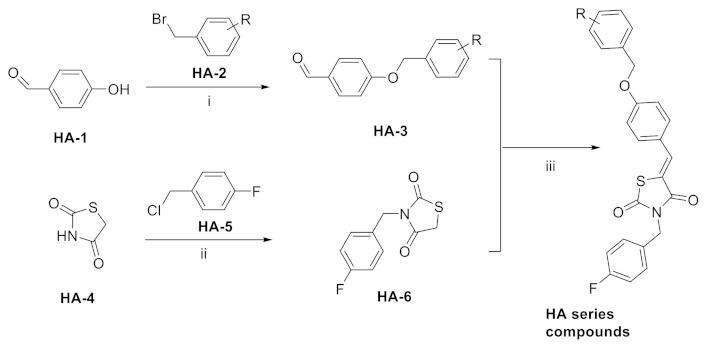
Synthesis of HA series compounds. Reagents and conditions: (i) KOH, DMSO, RT.; (ii) NaH, DMF, RT.; (iii) piperidine, EtOH, reflux.

The most classic acid head is boronic acid. In 2010, Harald et al. obtained the thiazolidinedione derivative HA51 (**22**) (IC_50_ = 1.07 μM) when using CPF4 for HTS. The synthesis of HA series compounds can be seen in Scheme 1 [62]. Inspired by the interaction between the boronic acid group in the proteasome inhibitor bortezomib and threonine hydroxyl oxygen in the proteasome HA51, carboxylic acids were changed to boronic acid to obtain HA130 (**23**) (IC_50_ = 28 nM, LPC). The co-crystal structure indicated that boronic acid in HA130 interacted with Thr209 [21], thus improving the potency compared with carboxylic acid derivatives. The intravenous administration of HA130 (1 nmol/g) to mice rapidly decreased plasma LPA concentration and dose-dependently inhibited ATX-mediated migration of A2058 human melanoma cells. However, it had very poor metabolic stability (t_1/2_ < 5 min) [62,63]. Subsequent ATX structure-based optimization of this series of compounds by this group revealed that boronic acid in the para position in HA130 could improve potency; that is, HA155 (**20**) (IC_50_ = 5.7 nM, LPC). The co-crystal structure indicated that HA155 interacted with Thr209, Asp311, His359 and His474 at the active site and 4F-Bn guided by thiazolidinediones interacted with Leu213 and Phe274 at the hydrophobic pocket. However, the in vivo stability of HA155 was still very low, possibly due to potential Michael receptors in this series of compounds [64]. In 2013, Mitsuyasu et al. performed HTS on 81,600 compounds using the highly sensitive and specific fluorescent probe TG-mTMP developed by this group to obtain the hit compound **24** (IC_50_ = 180 nM, TG-mTMP). The co-crystal of **24** and ATX indicated that **24** formed a hydrogen bond between the nitrogen atom of the thiazole ring and Trp275 without interacting with the zinc ions and Thr209 in the active site, and the tail did not fully penetrate into the hydrophobic pocket. The optimization was to target the zinc ions and Thr209 and extend into the pocket in order to obtain a tighter binding to ATX. Finally, 2BoA (**25**) (IC_50_ = 580 nM, TG-mTMP), 3BoA (**26**) (IC_50_ = 13 nM, TG-mTMP), and 4BoA (**27**) (IC_50_ = 22 nM, TG-mTMP) were obtained, showing that the position of the boronic acid substituent directly affected whether the inhibitor interacted with the catalytic center. The synthesis of BoA series compounds is similar to the HA series compounds. ATX-induced cell motility assay was performed using MDAMB-231 cells, all three compounds inhibited cell motility in a dose-dependent manner, and mouse plasma LPA levels decreased to almost zero after 10 min of administration (4 mg/kg) of 3BoA [65]. Recently, Kraljic et al. used benzoxaboroles to replace boric acid. The annular tension made the boron atom have a stronger Lewis acid activity and facilitate the binding to Thr209. At the same time, Kraljic et al. switched the corespacer of HA155 to a more stable and rigid fragment to improve ADME properties, through which Compound **28** (IC_50_ = 130 nM, LPC) was obtained. Although the potency of **28** was decreased compared with HA155 (IC_50_ = 88 nM, LPC), the solubility was 30 times higher than HA155 (30–100 μM), and the metabolic stability of liver microsomes was improved (62%/85%, percentage of hepatic blood flow% LBF, mATX/hATX) [66]. Boronic acid structures are also present in many other ATX inhibitor patents, such as carbazole compound SW-A3 (**29**) (IC_50_ = 33.8 nM, FS-3).

Apart from boronic acids, phosphoric and carboxylic acid heads, which are widely contained in lipid-like inhibitors, are also used in small molecule inhibitors. Examples include phenylthiazole inhibitor **30** (IC_50_ = 2.19 nM, FS-3), with a phosphoric head developed by Balupuri et al. Ono Pharmaceutical Co. Ltd. obtained a hit compound **31** during HTS on an internal compound library. It was considered to lack a polar group compared with the ATX substrate LPC, so a carboxyl group with a linker of different lengths was introduced into the acetyl part and the phenyl ring. Compound **31** was optimized to obtain compound **32** (IC_50_ = 17 nM); however, **32** had poor inhibitory potency on ATX in plasma (IC_50_ = 0.26 μM), presumably due to its high plasma protein binding rate. Therefore, the rigidity around the carboxyl group was increased and isomerization was eliminated instead of eliminating the complexity of clinical studies. Finally, compound **33** was obtained (IC_50_ = 10 nM/55 nM, hATX/plasma assay). In vivo studies showed that compound **33** could enhance the anti-tumor effect of paclitaxel in mouse models of breast cancer cell lines [67]. Recently, inspired by the structure of S32826, Nikolaou et al. introduced isotaximic acid, a zinc ion-binding group, to **33**. Starting with hydroxamic acid containing 4-aminophenylacetic acid residues, they obtained compound **34** (IC_50_ = 60 nM, LPC) after SAR studies on linker length, necessity of isotaximic acid, and amino acid substitution in the 4-aminophenylacetic acid part. Molecular docking results showed that hydroxamic acid interacted with Asn230, Tyr306 and Thr209. After intraperitoneal injection (**35** ng/mL) of **34** to a mouse model of bleomycin-induced pulmonary inflammation and fibrosis (C57BL/6) for 15 days, no significant toxicity was observed during the experiment, and a high concentration of **34** was also detected in mouse plasma after 6 h of administration [68].

In addition to the common acidic groups mentioned above, heterocyclic compounds with certain acidity and alkalinity such as tetrazolium (a bioelectron isostere of carboxylic acid), triazole, imidazole and benzoxazolone can also bind to the active site. Jones explored the SAR of heterocyclic groups of aminopyrimidines binding to zinc ions and then proposed a possible relationship between the pKa value of the binding groups and binding ability; that is, for acidic heterocycles, in order to maximize binding ability, the heterocycles are deprotonated only when bound to zinc ions, and will impose a higher desolvation penalty if preionized (i.e., too acidic, e.g., pKa < ~7.4), and will be unable to be deprotonated at physiological PH if pKa is too high (e.g., pKa > ~11). Correspondingly, there is no obvious increase in the binding energy of the system when the ideal basic binding group can bind to zinc ions, that is to say, it is mainly in the state of electrical properties (pKa < ~8) at physiological PH. Of course, it is not conducive to binding to zinc ions if the pKa is too low [71]. In addition to expanding the optional range of head structure, the common acidic groups are more conducive to regulating the overall lipid and water distribution coefficient of the molecule. Therefore, many ATX inhibitors have this type of structure, which will be mentioned in the subsequent introduction.

In summary, the main function of the head part is to interact with zinc ions and amino acid residues such as Thr209 at the active site in order to improve the binding efficacy of inhibitors with ATX. However, as such, excessive interaction with zinc ions may have a risk of off-target action, and reduce the selectivity of inhibitors. Of course, this also provides a certain opportunity. For example, leukotriene A4 hydrolase (LTAH) inhibitor **35**, recently reported, also has isotaximic acid, with some similarity to the structure of compound **34**; **35** can also reduce the inflammatory response in the early stage of IPF in terms of pharmacological effects [69], indicating that ATX inhibitors can be considered to develop dual or multiple inhibitory functions, thereby improving efficacy for a certain disease.

#### 5.2.2. Tail Anchored at the Hydrophobic Pocket

**Scheme 2:**
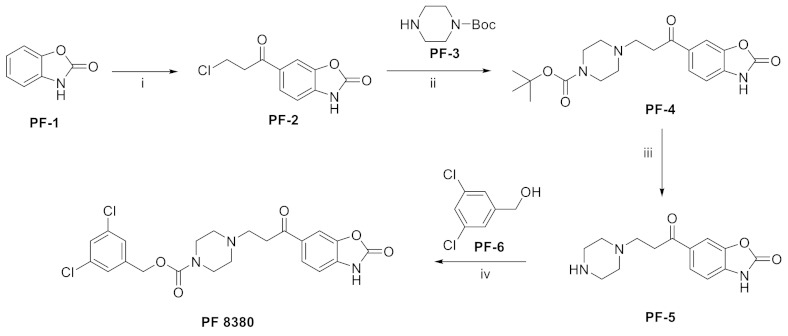
Synthesis of PF8380. Reagents and conditions: (i) AlCl_3_, DMF, RT.; (ii) Et_3_N, DCM, RT.; (iii) 4N HCl, dioxane, CH_3_CN, RT.; (iv) 1, 1′-carbonyldilmidazole, DCM, RT.

We have previously mentioned the structure of the ATX hydrophobic pocket, and it is easy to speculate that the tail needs to have some hydrophobicity. The most classical tail structure is the 3, 5-dichlorobenzyl group in PF8380 (**21**); PF8380 (IC_50_ = 1.7/101 nM, LPC/plasma assay, the synthesis route of PF8380 is shown in Scheme 2 [21]) was discovered by Pfizer during the development of new anti-inflammatory drugs in which the benzoxazolone head interacts with a zinc ion and is close to Thr209, while the hydrophobic tail has hydrophobic interactions with Leu213, Phe273 and Phe274. At an oral dose of 100 mg/kg, the plasma ATX inhibitor can reach 95%, and the human total EC_50_ value is about 0.08 μM, which can dose-dependently reduce hyperalgesia. It was the first ATX inhibitor to reduce the concentration of LPA in the body and has good kinetic characteristics, with an effect similar to that of the non-steroidal anti-inflammatory drug Naproxen [74]. Other studies have shown that PF-8380 attenuates leukomycin-induced pulmonary fibrosis and attenuates cardiac dysfunction and inflammatory response induced by a high-fat diet in mice [75]. In 2017, Kuttruff et al. showed in the study that PF8380 had poor metabolic stability in liver microsomes and an excessive inhibitory effect on hERG (IC_50_ = 480 nM). In order to solve these problems, this group changed the piperazine core in PF8380 and finally obtained BI2545 (**36**) (IC_50_ = 2.2/29 nM, LPC/plasma assay) after SAR optimization of linker, head and tail. From the structural point of view, BI2545 still used the 3, 5-substituted benzene ring as the tail, and molecular docking also indicated that its binding mode was similar to that of PF8380. In vivo experiments showed that BI2545 had good stability in human and mouse liver microsomes, good oral availability and low plasma clearance in mice, and the clearance rate of LPA could reach more than 90%. In addition to this, the classical structure of PF8380 tail is also used in phenylthiazole, compound **30** [70].

In addition to the hydrophobic tail structures of various substituted benzene rings, 2-indanamine is also a commonly used structure. This structure (**37**) [71] was used in the development of PF8380 analogues by Eli Lilly in 2016. The key to the selection of the classical inhibitor tail structure is to form hydrophobic interactions with the corresponding amino acid residues at the hydrophobic pocket. Since the focus is often placed on the core spacer in the design process of the ATX inhibitor core structure in order to improve the druggability of the molecule, the above simpler hydrophobic tail structure is often used in order to provide a modification space for the core spacer. Of course, the type of tail or the choice of various substituent positions and types on the aromatic ring should take the orientation and physicochemical properties of the overall molecular structure into account.

#### 5.2.3. Core Spacer

Compared with head and tail, the core spacer is a key aspect in the design of ATX inhibitors. The study of this part has a very important impact on the novelty of drug structure, the rigidity of its structure, the improvement of binding efficacy and the overall orientation of the molecule and its interaction with ATX. Different research groups commonly use HTS screening to obtain a better advantage structure or pharmacophore and, combining the cocrystal structure of PF8380 or HA155 with ATX, select the appropriate head and tail to extend the molecule so as to achieve the best pose. The main modification site of many ATX inhibitors also lies in this.

**Scheme 3:**
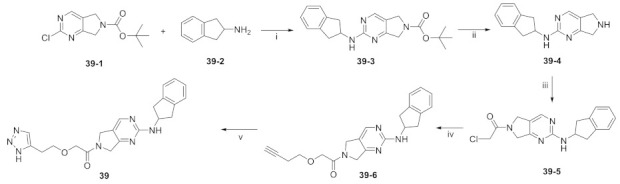
Synthesis of 39. Reagents and conditions: (i) iPr_2_EtN, NMP, 80 °C; (ii) 5N HCl, THF, 50 °C; (iii) 2-chloroacetyl chloride, Et_3_N, DCM, RT.; (iv) 3-butyn-1-ol, NaH, THF, 0 °C; (v) CuSO_4_ 5H_2_O, L-sodium ascorbate, TMSN_3_, DMF, H_2_O, 90 °C.

In 2016, Eli Lilly yielded a hit compound **37** through HTS with a tetrahydropyrimidine structure (IC_50_ = 520/1070 nM, LPC/plasma assay), and the co-crystal structure of **37** with ATX showed that the pyrimidine part bound to the pocket and had π-π interactions with Phe273, N and NH had hydrogen-bond interactions with Trp275 and Phe273, and the scaffold had π-π interactions with Tyr306. The extension to active site was performed on **37**, and the benzoxazolone structure was introduced to obtain **38** (IC_50_ = 2.5/42 nM, LPC/plasma assay); however, **38** had problems such as poor solubility, high metabolism in liver microsomes, and poor PK value in rats, which hindered further development. Triazole and imidazole were considered to replace benzoxazolone to bind to Zn2+ and the length and type of linker were optimized, and compound **39** was finally obtained (IC_50_ ≤ 1.7/2.2 nM, LPC/plasma assay; the synthesis route of **39** is shown in Scheme 3 [71]). The co-crystal structure indicated that **39** interacted with Asp171, Asp311, His315 and His474 in the active site in addition to zinc ions, similar to PF8380, as well as with Leu213, Phe273, Phe274, Trp275 at the pocket. Compound **39** has a good PK/PD relationship and can be used to study the role of ATX in osteoarthritis pain [71]. Gerokonstantis et al. used the advantage scaffold 2-pyrrolidone that appears in many drugs (piracetam, doxapram, etc.) as a core in order to perform SAR analysis on carboxylic acids, esters, sulfonamides, boric acids, etc. on the basis of S-pyroglutamic acid to obtain 2-pyrrolidone, compound **40** (IC_50_ = 35 nM, LPC), and pyrrolidine, compound **41** (IC_50_ = 800 nM, LPC);compound **40** interacted with Thr209 and Asn230, with binding energies similar to HA155 [72]. In addition, phenylthiazole compounds also have many different physiological activities, such as anticancer and antifungal activities. Balupuri et al. used 4-phenylthiazole as a core to connect a phosphate head with a piperidine tail in PF8380 to obtain compound **30** (IC_50_ = 2.19 nM, FS-3), which also has high potency in human plasma (IC_50_ = 14.99 nM, plasma assay) [76]. In order to make this series of compounds have better druggability, the group subsequently continued to optimize compound **30**. Since the linker of this compound did not significantly interact with ATX, it was decided to modify this part first, finally obtaining compound **42** (IC_50_ = 1.23/2.18 nM, FS-3/bis-pNPP), which showed strong anticancer effects in liver cancer and melanoma cell lines (EC_50_ = 7.90 and 1.30 nM) in cell experiments. In vivo experiments showed that **42** showed very promising kinetic characteristics after intravenous injection in rats, and metabolism was also stable in human and rat liver microsomes [73]; however, the highly polar and highly charged characteristics of phosphate groups were not suitable for oral administration and could be further modified.

In summary, such classical inhibitor types have clear methods of design and high binding efficacy now that the three-dimensional structure of ATX crystals is disclosed, and include the very potent HA155 and PF8380.Unfortunately, no inhibitor of this type successfully reached the level of clinical experiments. It is assumed that there may be some off-target effect by acting on the active site; also, such compounds often contain acidic groups, and their physicochemical properties are difficult to control in terms of druggability. Of course, it is more likely that our understanding of the ATX-LPA axis is simply not complete; in the physiological processes related to ATX, the downstream pathological reactions caused by LPA decomposing LPC account for only a small part of LPA pathogenesis, and other functions of ATX such as transport of LPA to LPAR may also be very important; thus, it is very important to deeply understand the functions of each part of the ATX PDE domain for the further development of ATX inhibitors.

## 6. Allosteric Inhibitors

When the co-crystal structure of ATX and ligand was solved, the main idea of the binding mode of ATX inhibitor to ATX was to occupy the same site as LPC, resulting in the above classic inhibitor, which was also a type I inhibitor. The main ways of discovering new inhibitors are rational optimization, brand-new design based on ATX structure and existing inhibitors, or structural modification of the resulting hit compound using HTS. During this process, it was found that some of the screened compounds with inhibitory effects and ability to bind to ATX were not at the orthosteric site (type I inhibitors), but possessed a new binding mode, that is, away from the active site, and formed uncompetitive inhibition only at the hydrophobic pocket (type II) or at the tunnel (type III) located at the allosteric site (Figure 2B,C). Inhibitors located in the pocket can hinder the binding of LPC to ATX, and inhibitors located in the tunnel can hinder the release and transport of LPA produced by ATX hydrolysis. It can be speculated that these binding modes can more widely inhibit the effect of ATX, and due to the distance from the active site containing zinc ions, selective inhibitors can be obtained.

**Scheme 4:**
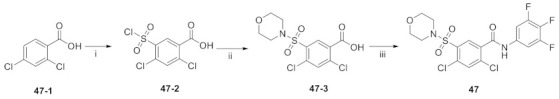
Synthesis of 47. Reagents and conditions: (i) ClSO_3_H, reflux; (ii) morpholine, Et_3_N, RT; (iii) (a) SOCl_2_, DMF (cat.); (b) substituted-aniline pyridine, RT.

In 2013, when HTS was performed by Fells et al., **43** (compound **43**–**62** in Table 4) (IC_50_ = 43.6 nM, LPC), **44** (IC_50_ = 57.6 nM, LPC) and **45** (IC_50_ = 78.2 nM, LPC) were obtained by primary screening with FS-3 as substrate and rescreening with pNP-TMP and ADMAN-LPC. When the artificial substrate was used for screening, it was found that **43** and **44** did not block the hydrolysis of pNP-TMP and produced false negative results, but inhibited the hydrolysis of FS-3, while **45** inhibited the hydrolysis of both FS-3 and pNP-TMP. In order to explore the reasons for this, **43**, **44**, **45** and pNP-TMP were molecularly docked with ATX, and the results showed that pNP-TMP bound to the catalytic site of ATX; **43** penetrated into the hydrophobic pocket, and the morpholine ring was located at the entrance of the tunnel; **44** penetrated into the pocket and tunnel; and **45** occupied with the tunnel and catalytic site, which explained why **43** and **44** had false negative results. Cell experiments showed that all three inhibited the invasion of melanoma A2058 cell line, with an EC_50_ of 118.79, 153.05, and 85.35 nM, respectively; intravenous injection of **43** into C57BL/6 mice also inhibited the metastasis of melanoma cells B16-F10 in vivo, demonstrating that this compound bound to the hydrophobic pocket can also play a blocking role [77]. Although this group did not perform co-crystal structure analysis of inhibitor and ATX, this is the first reported inhibitor away from the catalytic site. Subsequent SAR analysis of **43** (IC_50_ = 117 nM, FS-3) by this group yielded compound **46** (IC_50_ = 21 nM, FS-3) with competitive inhibitor Ki = 8.4 nM. Molecular docking results showed that it was similar to **43**. Phenylsulfonamide had π-π interactions with Phe211, Tyr307 and Phe275, of which Phe275 was very important in its binding to ATX through its effect on Phe275 mutants [78]. Due to the lack of high potency and in vivo stability of **43** and **46**, **47**  (the synthesis route of **47** is showed in Scheme 4 [79]) was synthesized by this group in 2017. This compound is a dual inhibitor of ATX and LPA1, with IC_50_ of 9 nM and 14 μM, respectively, and can inhibit the invasion of A2058 in a dose-dependent manner (EC_50_ = 528 nM) [79]. Recently, in order to improve the potency of this series of compounds in dual inhibition of ATX and LPAR, and therefore overcome LPA-mediated resistance to chemotherapy and radiotherapy, this group used **47** and ATX and LPA1′s dual inhibitor, BrP-LPA (IC_50_ = 800/273 nM, ATX/LPAR1), as well as ATX and LPA1′s structures to conduct QSAR. They analyzed the interaction site between LPAR1 and its inhibitors, then docked **46** with LPAR1 and modified **47** to obtain compounds **48** and **49**. Unfortunately, these two compounds did not inhibit LPAR1; however, it was a positive attempt to develop new inhibitors with QSAR using ATX inhibitors [80].

The first ATX inhibitor with a new binding mode possessing a cocrystal structure was a PAT series of compounds developed by PharmAkea in 2015. They performed HTS using FS-3 and found four indole derivatives, PAT-078 (**50**) (IC_50_ = 472 nM, LPC), PAT-494 (**51**) (IC_50_ = 20 nM, LPC), PAT-352 (**52**) (IC_50_ = 26 nM, LPC), and PAT-347 (**53**) (IC_50_ = 0.3 nM, LPC). The cocrystal structure showed that PAT-078 occupied the hydrophobic pocket, the vinyl nitrile moiety interacted with Tyr307 to form p-p and H-bonds with Phe275 amide; PAT-494 partially occupied the tunnel entrance, the acetonylurea moiety interacted with Tyr307 to form π–π and H-bonds with Phe275 amide; PAT-352 had two binding modes with ATX, one extending to the catalytic site, and the other extending to the tunnel; and benzoic acid partially has π–π interactions with Phe249 but also with Lys248, Trp254, and Trp260. As for why molecules bound to the tunnel inhibit the catalytic effect of ATX, the authors gave the explanation that this inhibitor does not affect the cleavage of LPC, but prevents the generated LPA from exiting the enzyme through hydrophobic channels, thereby preventing the binding of ATX to the next protein [81]. PharmAkea also obtained two other compounds with the same indole ring, PAT-505 (**54**) (IC_50_ = 2 nM, LPC) and PAT-048 (**55**) (IC_50_ = 1.1 nM, LPC), which were also very potent in human plasma, with IC50 of 9.7 nM and 8.9 nM, respectively; however, PAT-048 was ineffective in a bleomycin-induced pulmonary fibrosis model. PAT-505 showed antifibrotic effects in a mouse model of NASH with favorable PK/PD properties, and the co-crystal structure showed that PAT-505 bound in a similar pattern to PAT-347 and interacted with Lys248, Phe249, His251, Trp254, Trp260 and Phe274 [82]. Miller et al. analyzed the SAR of **56** (IC_50_ < 300 nM, LPC) of PharmAkea PAT series compounds in 2017, including chlorine substitution at the 6-position of the indole ring, indole N substitution, and thiopyridinecarboxylic acid substitution. Finally, the optimized compound **57** (IC_50_ = 81 nM, LPC) was obtained, with good water solubility and excellent LLE [83].

After the discovery of the above benzene sulfonamide and PAT series compounds, more and more attention has been paid to whether compounds bind to the pocket or tunnel. In 2016, Shah et al. performed HTS of 87,865 compounds using FS-3, followed by rescreening with LPC to obtain hit compound **58** (IC_50_ = 473 nM, LPC). After SAR analysis and optimization of benzene ring, linker and pyrimidine pyridine core, compound CRT0273750 (**59**) (IC_50_ = 1/14 nM, LPC/plasma assay) was obtained. The molecular docking results showed that 59 went deep into the pocket and bound to the tunnel edge, interacting with Leu213, Phe248, Trp254, Phe273/274 and Trp275. The properties of **59** were also very good; the oral bioavailability in mice was 41%, and the half-life reached 5.4 h in SD rats. Pharmacological experiments showed that this compound inhibited the migration of 4T1 cells, and also inhibited the 18:1 LPA level in human plasma in vitro and in vivo samples from MDA-MV-231-luc tumor mice [84]. In 2017, Pantsar et al. used the co-crystal structures of mATX with HA155 and PF8380 as models to perform virtual screening of 2.7 million compounds, and then used ewATX (hen egg white) to determine 26 potential compounds and found that two compounds possessing 2, 4-dihydropyrano [2, 3-c] pyrazole scaffold had ATX inhibitory activity. The preliminary SAR showed that the presence of benzene ring on the pyrazole ring was important and only the S conformation was active; thus the substitution, mainly on the benzene ring, was modified to obtain compound **60** (ewATX/hATX IC_50_ = 87/134 nM, LPC). The molecular docking results showed that **60** binds to the pocket and was verified with the artificial substrate pNP-TMP. The results demonstrated that **60** could not completely inhibit the effect of pNP-TMP. In vitro cell experiments showed that **60** inhibited the migration of melanoma cells A2058 induced by ATX, but had no effect on migration induced by LPA. In addition to developing new inhibitors, this group is interested in expanding the use of ewATX. The amino acid sequences of human and henENPP2 have 83% identity, and from the pocket amino acid residues only Leu260 is replaced by Ile260; therefore, ewATX has a pocket highly similar to hATX and can be used as an initial screening test for ATX inhibitor discovery. Final validation with the results of hATX showed that the active compounds showed only a small difference (mean 2.68%) in the degree of ewATX and hATX inhibition, except for the four outliers [85].

In addition to synthetic ATX inhibitors, Keune et al. found there was steroid binding to the tunnel when studying the catalytic mechanism of ATX. Subsequent studies showed that 7HCS (**61**, 7-α-cholesterol) did not inhibit the hydrolysis of ATX; however, TUDCA (tauroursodeoxycholic acid) (**62**) and UDCA (urosodeoxycholic acid) could inhibit ATX activity, with IC_50_ of 10.4 and 8.8 μM (LPC), respectively. Molecular docking showed that TUDCA formed a hydrogen bond with Trp260 and had hydrophobic interaction with Trp254 and Phe274. The group speculated that UDCA may cause pruritus caused by cholestasis by interfering with the ATX-LPA axis [23].

These small molecule structures expand the diversity of ATX inhibitors and provide ample tools for introgression studies of the ATX catalytic site, pocket, and tunnel functions.

## 7. GLPG1690-Based ATX Inhibitors

**Scheme 5:**
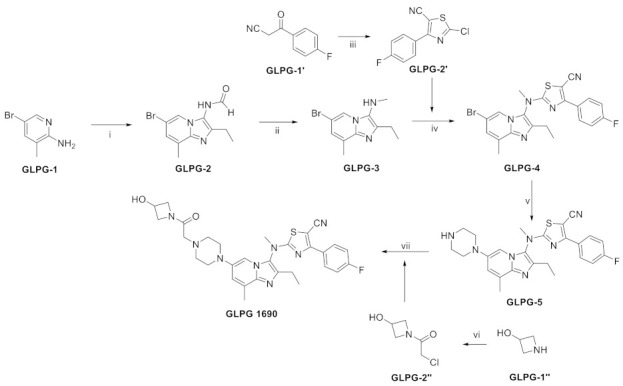
Synthesis of GLPG1690. Reagents and conditions: (i) (a) EtCHO, benzotriazole, toluene, RT., then KCN, EtOH, RT. to 80 °C; (b) AcCl, EtOH, RT. (c) HCO_2_H, 80 °C; (ii) (a) K_2_CO_3_, MeI, acetone, 80 °C, (b) HCl, MeOH, 80 °C; (iii) (a) pyridine, thiourea, iodine, EtOH, 70 °C to RT.; (b) CuCl_2_, ^t^BuONO, MeCN, RT; (iv) (a) NaH, THF, reflux; (b) GLPG-2′, 40 °C to reflux (v) (a) Boc-piperazine, tBuONa, Pd_2_dba_3_, JohnPhos, toluene, 115 °C; (b) HCl, MeOH, RT.; (vi) chloroacetyl chloride, K_2_CO_3_, H_2_O, DCM, RT.; (vii) GLPG-2′’, K_2_CO_3_, MeCN, reflux.

GLPG1690 (**63**) (compound **63**–**83** in Table 5; the synthesis route of GLPG1690 is shown in Scheme 5 [86]), an ATX inhibitor developed by Galapagos for the treatment of IPF in 2014, was the first ATX inhibitor to enter clinical trials, thus showing the advantages of its structure and good kinetic properties. In 2017, its development process was revealed. Galapagos performed HTS screening of an internal library of molecules using FS-3 as a substrate to obtain a hit compound series possessing the same structure as **64**, which was analyzed by SAR to obtain compound **65** (IC_50_ = 27/22 nM, LPC/plasma assay); the co-crystal structure indicated that **65** formed hydrogen bonds with Trp260 and had hydrophobic interactions with Phe250, 260, 275 and Trp254. However, **65** has limited oral exposure in mice, inhibits hERG channels (IC_50_ = 2.9 μM), and has a time-dependent inhibitory effect on cytochrome P450 in human liver, which is not conducive to clinical development [87]. To ameliorate these issues, they considered adding a methyl group to the scaffold to prevent oxidation and replacing piperidine with piperazine to reduce inhibition of hERG, resulting in GLPG1690, with an IC_50_ of 131, 418, 542, 242 nM and 15 μM for LPC, mouse plasma, rat plasma, human plasma, and hERG, respectively. The co-crystal structure indicates that the key is in the tunnel with the N atom of piperazine forming a hydrogen bond with Trp255, the acetyl chain with Trp261, and the tail forming a phenyl ring interaction with Phe274 [87]. This mode of action with pocket and tunnel becomes another new binding mode (Figure 2D); due to the success of GLPG1690 in clinical trials the advancement of this binding mode is also illustrated to some extent, and many subsequent groups are also developing new ATX inhibitors based on the mode of GLPG1690.

There are often two ways to develop such compounds. The advantage scaffold obtained by HTS screening is simulated and modified based on the pose of GLPG1690 in ATX. The other is to combine the hydrophobic tail part of type I inhibitor with the inhibitor acting on tunnel to obtain a binding mode similar to GLPG1690. Keune’s group, which found that TUDCA could bind to ATX tunnel, hybridized TUDCA with PF8380 to obtain compound **66** (IC_50_ = 20 nM, LPC), which was a competitive inhibitor (Ki = 6 nM), and the co-crystal structure with rATX indicated that **66** formed hydrogen bonds with Tyr81 and Trp260 at the channel, and formed π-π stacking with Trp274 and Phe273 at the hydrophobic pocket. In vivo experiments showed that after intravenous injection in mice (10 mg/kg), the degree of effect in vivo was similar to that of PF8380, and LPA levels could still be regulated 8 h after administration [88].

Zhai et al. had previously developed EGFR inhibitors with a tetrahydropyrimidine scaffold. Based on the above-mentioned Eli Lilly and Pfizer developed structure (compound **40**), they developed EGFR and ATX dual inhibitors on this tetrahydropyrimidine scaffold considering the key role of EGFR in lung fibrosis caused by lung cancer, and obtained compounds **67** (IC_50_ = 38.4/42.3 nM, ATX/EGFR) and **68** (IC_50_ = 29.1/24.2 nM, ATX/EGFR). The molecular docking results indicated that compound **67** formed two hydrogen bonds with Ala218 and Trp276, specifically, the tetrahydropyrimidine scaffold attached to Leu214, Phe274 and Tyr307 through hydrophobic interactions, while forming hydrophobic interactions with the hydrophobic pocket. Cell experiments have shown that **68** has an effect on cardiac fibroblasts (CFs) and RAW264.7 macrophages and can be used as a potential dual inhibitor of EGFR and ATX in the treatment of pulmonary fibrosis caused by lung cancer [89]. Subsequently, this group optimized **68** for SAR and obtained compound **69** (IC_50_ = 24.2 nM, LPC) and 70 (IC_50_ = 15.3 nM, LPC) with a similar scaffold to GLPG1690, both of which had similar binding modes to 68. Cell experiments showed that **69** had a greater advantage in CFs, with a maximum inhibition rate of 81.5%, and **70** had a significant advantage in hepatic stellate cells (HSCs), with a maximum inhibition rate of 83.7%, which is worthy of further evaluation for cardiac and pulmonary fibrosis, respectively [90]. After understanding the great potential of ATX as a drug target Zhai’s group recently performed HTS using FS-3 as a substrate to obtain indole compound **71** as hit compound (IC_50_ = 740 nM, FS-3). First, they considered the use of acylhydrazine as a linker to guide the aromatic ring deep into the pocket, but it did not achieve the ideal effect. Then, followed by amide reversal to form a carbamate linker, hydroxyl-containing piperazine and piperidine were introduced, and finally compounds **72** (IC_50_ = 1.01 nM, FS-3) and **73** (IC_50_ = 0.43 nM, FS-3) were obtained. Comparison with the co-crystal structure of GLPG1690 revealed that the carbamate linker ideally bridges the indole scaffold and aromatic groups to the hydrophobic pocket and forms a necessary H-bond with the Phe274 residue (carbamate−NH in **73**). At the same time, the hydroxyl-containing piperidine occupies part of the tunnel well, and the hydroxyl group in **73** also forms an additional H-bond with the Gly256 residue. Using GLPG1690 as a control, **72** and **73** were evaluated in a BLM-induced lung fibrosis model, both of which were potentially protective for the model, and **73** was more protective than GLPG1690 in vitro [91]. At the same time, this group also designed compounds **74** (IC_50_ = 2.1 nM, FS-3) and **75** (IC_50_ = 1.9 nM, FS-3) by hybridization with the phenylthiazole moiety of GLPG1690 using acylhydrazine as a linker, both of which inhibited the proliferation of breast cancer cells MCF-7 and MDA-MB-231 cells [92], as well as **76** (IC_50_ = 2.3 nM, FS-3), using urea as a linker [93].

Kawaguchi et al. used TG-mTMP, developed by their group as a probe, performed HTS on compounds bound to pocket and tunnel to obtain hit compound **77** (IC_50_ = 4.2 nM, TG-mTMP); the co-crystal structure indicated that **77** bound to the hydrophobic pocket with its carbonyl group forming a hydrogen bond with the main-chain amide group of Trp275, with the ethyl acetate part interacting with the hydrophobic channel formed by Phe210, Tyr214, Trp254, Trp260, and Phe274. However, the mouse plasma ATX of **77** had a poor inhibitory effect, and it was speculated that **77** was unstable in plasma (**77** was inactivated after hydrolysis into a carboxyl group). In order to improve the problem, SAR analysis of the imidazolopyrimidinone scaffold, benzene ring substitution and ethyl acetate site showed that the substitution of a hydrophobic structure at position 8 on the imidazolopyrimidinone scaffold was very important for its ATX inhibitory effect, because this scaffold will form an enol structure in solution and cannot form a hydrogen bond with Trp275. Adding a hydrophobic structure at position 8 can fix it in the form of ketone and interact with Trp275. Finally, metabolically stable compounds **78** and **79** were obtained; **79** could dose-dependently inhibit cell motility in ATX-induced cell motility assay in MDA-MB-231 cells, with an IC_50_ of 30 nM. In vivo experiments in mice showed that LPA rapidly decreased within 15 min after injection of **78** and **79** and remained at a low level for 3 h [94].

In recent years, due to the accumulation of a large number of structurally diverse ATX inhibitors, we can also use other data analysis methods such as virtual screening and QSAR for the development of new ATX inhibitors in addition to HTS. Banerleadjee et al. established and validated 14 pharmacophore models using the receptor ligand complex of GLPG1690 and lead compound **64**, and then used this pharmacophore model for virtual screening to obtain nine compounds with similar binding modes to GLPG1690 [80]. Ren et al. used the co-crystal structure of ATX and HA-155 as the molecular model to perform virtual screening of 144,000 compounds in the HitFinder database and in vitro enzymatic screening to obtain KM03601 and SCR01013 with a certain potency. On the same basis, analogue screening was performed to obtain six new inhibitors, KM-14, KM-24, KM-26, KM-28, SC-41 and SC-49, of which KM28 (**80**) (IC_50_ = 1.8 μM, LPC) and SC-49 (**81**) (IC_50_ = 10.2 μM, LPC) were the best, which is worthy of further optimization with novelty in structure [95]. Lee et al. analyzed the topological water networks (TWNs) of ATX binding sites and provided new auxiliary ideas for the design of ATX [96]. Of course, there are also cases where the results do not match our expectations when performing ATX inhibitor designs. For example, when Thomson performed HTS with FS-3 based on PF-8380, **82** (IC_50_ = 9.6 nM, FS-3) with cinnamic acid structure was obtained, but its rat and human microsomal clearance rates were high (510 and 230 μL/min/mg, respectively). To improve the problem, SAR analysis was performed, and finally **83** (IC_50_ = 8 nM, LPC) was obtained; the clearance rate in rat liver microsomes was 15 μL/min/mg. The authors originally designed on the basis of a type I inhibitor, assuming that the tetrazolium moiety penetrates deep into the Catalytic site; however, the crystal structure suggests that piperazine guides the benzyl group into the tunnel and that the cinnamic acid moiety is located in the hydrophobic pocket [97]. This suggests that we are not able to take the designed compounds for granted and should fully study them, which will also aid in obtaining a deeper understanding of the structure and function of ATX.

In summary, from the results of current relevant studies and clinical trials, type IV inhibitors similar to GLPG1690 seem to be most promising in the development of ATX inhibitors, because such inhibitors can not only block the binding of LPC in the hydrophobic pocket of ATX but also block the function of the ATX tunnel part. That is to say, they can more comprehensively inhibit the function of ATX, and many new inhibitors are now continuously designed based on this. Of course, we cannot simply determine which type of inhibitor is better, and more experimental results are needed to prove which binding mode is more preferred. However, the clinical trials for GLPG-1690 have been recently discontinued in February 2021 due to issues with its safety and potency.

## 8. Perspectives and Conclusions

Of all the ATX inhibitors, there is as yet no drug successfully approved by the FDA on the market, and the only drugs currently entering clinical trials are GLPG-1690 (phase III), for the treatment of IPF, BBT-877 (phase I), recently from Boehringer Ingelheim, and BLD-0409, developed by Blade Therapeutics for the treatment of NASH; the structure and binding mode of the latter two have not been disclosed. However, in more than a decade of research on the structure and function of ATX, the accumulation of a variety of ATX inhibitors coupled with computer-assisted designing will certainly accelerate the development of new drugs. We believe that in future design of ATX inhibitors, more attention should be paid to the improvement of the in vitro and in vivo potency of inhibitors and the optimization of their kinetic properties and the other functions of ATX, as well as to the development of dual or multiple inhibitors and of irreversible inhibitors. For example, in LPA-mediated diseases, the common idea is to block the production of LPA or to block downstream signaling. However, using only ATX type I inhibitors may not achieve high potency in blocking the LPA pathway produced by the ATX pathway in vivo. Researchers can also focus on the transport function of the ATX tunnel and develop allosteric site inhibitors to block the transport of LPA to its receptor while blocking the production of LPA, so as to achieve better in vivo effect. In the above, we mentioned the extensive effects of ATX in diseases and complex interactions with multiple other pathways. We can see that in some diseases, ATX can cause the development of a disease together with many other targets. For example, in LPA-mediated diseases, we can develop multiple inhibitors of ATX, PLA and PPARs. In IPF response, we can develop dual inhibitors of ATX and inflammation targets. These possibilities are partly due to the flexibility of the ligand binding site of ATX. It has been mentioned earlier that loss of ATX in adult mice does not cause any severe phenotypic defects, indicating that most ATX activity is dispensable in adult life; thus, the development of irreversible inhibitors to enhance the inhibitory effect could also be considered.

## Figures and Tables

**Table 1:** Compound **1**–**11**.

No.	Structure	Assay	Biological Data	Reference
**1**	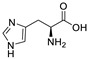	N.D.	N.D.	[46]
**2**	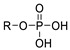	N.D.	N.D.	[47]
**3**	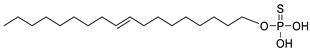	LPC assay	IC_50_ = 100 nM,	[48]
**4**	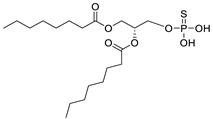	LPC assay	IC_50_ = 597 nM	[49]
**5**	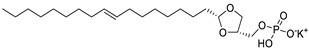	LPC assay	IC_50_ = 97 nM	[50]
**6**	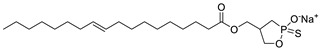	LPC assay	IC_50_ = 220 nM	[51,52]
**7**	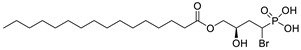	LPC assay	IC_50_ = 0.7–1.6 μM	[53]
**8**	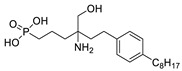	bis-pNPP assay	Ki = 0.2 μM	[54]
**9**	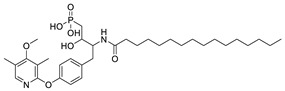	LPC assay	73% inhibition of ATX at 1 μM	[55]
**10**	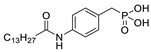	LPC assay	IC_50_ = 5.6 nM	[56]
**11**	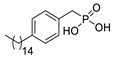	LPC assay	IC_50_ = 170 nM	[57]

**Table 2:** Compound **12**–**19**.

No.	Structure	Assay	Biological Data	Reference
**12**	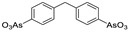	FS-3 assay	Ki = 240 nM	[59]
**13**	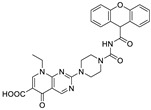	FS-3 assay pNP-TMP assay	>50% inhibition of ATX at 10 μM	[60]
**14**	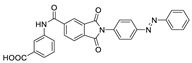	FS-3 assay pNP-TMP assay	>50% inhibition of ATX at 10 μM	
**15**	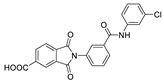	FS-3 assay pNP-TMP assay	>50% inhibition of ATX at 10 μM	
**16**	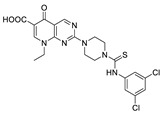	FS-3 assay	IC_50_ = 1.6 μM	[61]
**17**	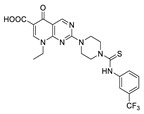	FS-3 assay	IC_50_ = 900 nM Ki = 0.7 μM	
**18**	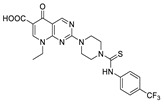	FS-3 assay	IC_50_ = 1.5 μM	
**19**	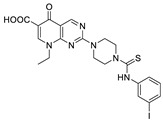	FS-3 assay	IC_50_ = 1.6 μM	

**Table 3:** Compound **20**–**42**.

No.	Structure	Assay	Biological Data	Reference
**20**	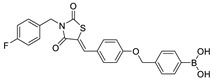	LPC assay	IC_50_ = 5.7 nM	[62]
**21**	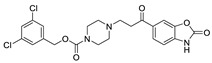	LPC assay	IC_50_ = 1.7 nM	[21]
**22**	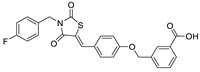	CPF4 assay	IC_50_ = 1.07 μM	
**23**	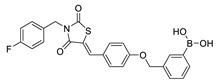	LPC assay	IC_50_ = 28 nM	
**24**	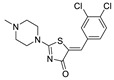	TG-mTMP assay	IC_50_ = 180 nM	[65]
**25**	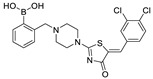	TG-mTMP assay	IC_50_ = 580 nM	
**26**	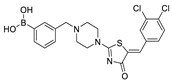	TG-mTMP assay	IC_50_ = 13 nM	
**27**	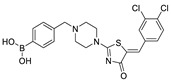	TG-mTMP assay	IC_50_ = 22 nM	
**28**	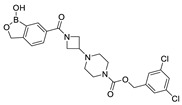	LPC assay	IC_50_ = 130 nM	[66]
**29**	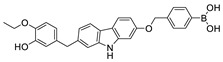	FS-3 assay	IC_50_ = 33.8 nM	CN107266356
**30**	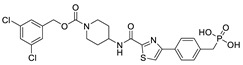	FS-3 assay	IC_50_ = 2.19 nM	[67]
**31**	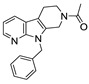	N.D.	N.D.	
**32**	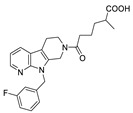	LPC assay Plasma assay	IC_50_ = 17 nM IC_50_ = 0.26 μM	
**33**	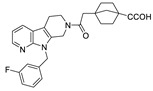	LPC assay Plasma assay	IC_50_ = 10 nM IC_50_ = 55 nM	
**34**	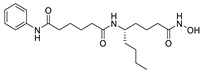	LPC assay	IC_50_ = 60 nM	[68]
**35**	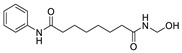	N.D.	N.D.	[69]
**36**	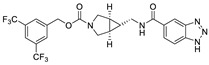	LPC assay Plasma assay	IC_50_ = 2.2 nM IC_50_ = 29 nM	[70]
**37**	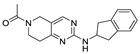	LPC assay Plasma assay	IC_50_ = 520 nM IC_50_ = 1070 nM	[71]
**38**	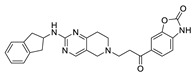	LPC assay Plasma assay	IC_50_ = 2.5 nM IC_50_ = 42 nM	
**39**	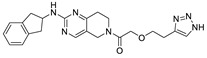	LPC assay Plasma assay	IC_50_ ≤ 1.7 nM IC_50_ ≤ 2.2 nM	
**40**	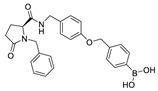	LPC assay	IC_50_ = 35 nM	[72]
**41**	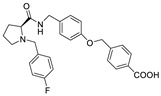	LPC assay	IC_50_ = 800 nM	
**42**	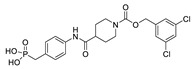	FS-3 assay bis-nNPP assay	IC_50_ = 1.23 nM IC_50_ = 2.18 nM	[73]

**Table 4:** Compound **43**–**62**.

No.	Structure	Assay	Biological Data	Reference
**43**	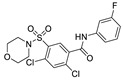	LPC assay	IC_50_ = 43.6 nM	[77]
**44**	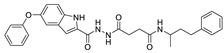	LPC assay	IC_50_ = 57.6 nM	
**45**	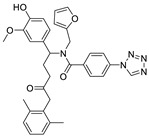	LPC assay	IC_50_ = 78.6 nM	
**46**	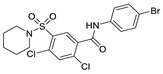	FS-3 assay	IC_50_ = 21 nM	[78]
**47**	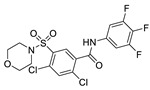	FS-3 assay	IC_50_ = 9 nM	[79]
**48**	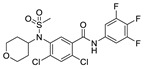	FS-3 assay	IC_50_ = 219 nM	[80]
**49**	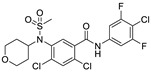	FS-3 assay	IC_50_ = 218 nM	
**50**	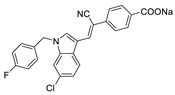	LPC assay	IC_50_ = 472 nM	[81]
**51**	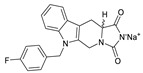	LPC assay	IC_50_ = 20 nM	
**52**	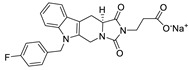	LPC assay	IC50 = 26 nM	
**53**	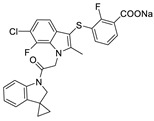	LPC assay	IC_50_ = 0.3 nM	
**54**	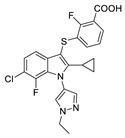	LPC assay Plasma assay	IC_50_ = 2 nM IC_50_ = 9.7 nM	[82]
**55**	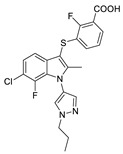	LPC assay Plasma assay	IC_50_ = 1.1 nM IC_50_ = 8.9 nM	
**56**	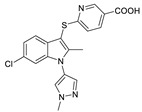	LPC assay	IC_50_ < 300 nM	[83]
**57**	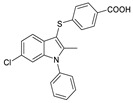	LPC assay	IC_50_ = 81 nM	
**58**	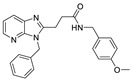	LPC assay	IC_50_ = 473 nM	[84]
**59**	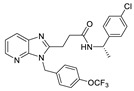	LPC assay Plasma assay	IC_50_ = 1 nM IC_50_ = 14 nM	
**60**	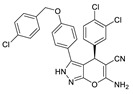	LPC assay(ewATX) LPC assay(hATX)	IC_50_ = 87 nM IC_50_ = 134 nM	[84]
**61**	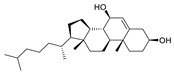	LPC assay	IC_50_ = 10.4 μM	[23]
**62**	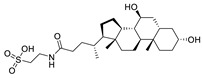	LPC assay	IC_50_ = 8.8 μM	

**Table 5:** Compound **63**–**83**.

No.	Structure	Assay	Biological Data	Reference
**63**	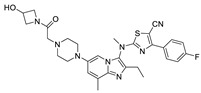	LPC assay Mice plasma assay rat plasma assay human plasma assay	IC_50_ = 131 nM IC_50_ = 418 μM IC_50_ = 542 nM IC_50_ = 242 μM	[86]
**64**	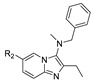	N.D.	N.D.	[87]
**65**	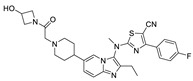	LPC assay Plasma assay	IC_50_ = 27 nM IC_50_ = 22 μM	
**66**	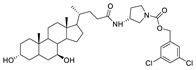	LPC assay	IC_50_ = 20 nM	[88]
**67**	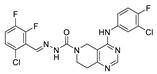	FS-3 assay	IC_50_ = 38.4 nM	[89]
**68**	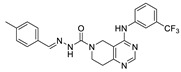	FS-3 assay	IC_50_ = 29.1 nM	
**69**	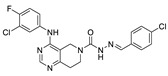	LPC assay	IC_50_ =24.2 nM	[90]
**70**	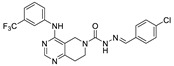	LPC assay	IC_50_ = 15.3 nM	
**71**	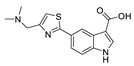	FS-3 assay	IC_50_ = 740 nM	[91]
**72**	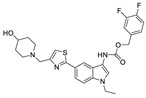	FS-3 assay	IC_50_ = 1.01 nM	
**73**	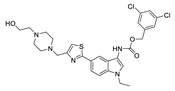	FS-3 assay	IC_50_ = 0.43 nM	
**74**	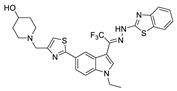	FS-3 assay	IC_50_ = 2.1 nM	[92]
**75**	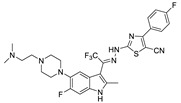	FS-3 assay	IC_50_ = 1.9 nM	
**76**	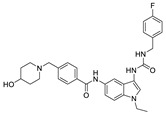	FS-3 assay	IC_50_ = 2.3 nM	[93]
**77**	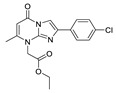	TG-mTMP assay	IC_50_ = 4.2 nM	[94]
**78**	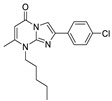	N.D.	N.D.	
**79**	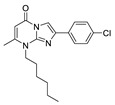	N.D.	N.D.	
**80**	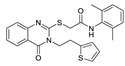	LPC assay	IC_50_ = 1.8 uM	[95]
**81**	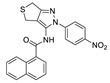	LPC assay	IC_50_ = 10.2 μM	
**82**	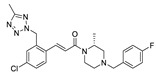	FS-3 assay	IC_50_ = 9.6 nM	[96]
**83**	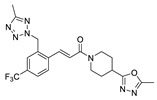	LPC assay	IC_50_ = 8 nM	

## Data Availability

Not applicable.

## References

[1] Stefan C., Jansen S., Bollen M. (2006). Modulation of purinergic signaling by NPP-type ectophosphodiesterases. Purinergic Signal..

[2] Stracke M.L., Krutzsch H.C., Unsworth E.J., Arestad A., Cioce V., Schiffmann E., Liotta L.A. (1992). Identification, purification, and partial sequence analysis of autotaxin, a novel motility-stimulating protein. J. Biol. Chem..

[3] Matralis A.N., Afantitis A., Aidinis V. (2019). Development and therapeutic potential of autotaxin small molecule inhibitors: From bench to advanced clinical trials. Med. Res. Rev..

[4] Tokumura A., Majima E., Kariya Y., Tominaga K., Kogure K., Yasuda K., Fukuzawa K. (2002). Identification of Human Plasma Lysophospholipase D, a Lysophosphatidic Acid-producing Enzyme, as Autotaxin, a Multifunctional Phosphodiesterase. J. Biol. Chem..

[5] Nakamura K., Kishimoto T., Ohkawa R., Okubo S., Tozuka M., Yokota H., Ikeda H., Ohshima N., Mizuno K., Yatomi Y. (2007). Suppression of lysophosphatidic acid and lysophosphatidylcholine formation in the plasma in vitro: Proposal of a plasma sample preparation method for laboratory testing of these lipids. Anal. Biochem..

[6] Jasinska R., Zhang Q.X., Pilquil C., Singh I., Xu J., Dewald J., Dillon D.A., Berthiaume L.G., Carman G.M., Waggoner D.W. (1999). Lipid phosphate phosphohydrolase-1 degrades exogenous glycerolipid and sphingolipid phosphate esters. Biochem. J..

[7] van Meeteren L., Moolenaar W. (2007). Regulation and biological activities of the autotaxin-LPA axis. Prog. Lipid Res..

[8] Tager A.M., LaCamera P., Shea B.S., Campanella G.S., Selman M., Zhao Z., Polosukhin V., Wain J., Karimi-Shah B.A., Kim N.D. (2008). The lysophosphatidic acid receptor LPA1 links pulmonary fibrosis to lung injury by mediating fibroblast recruitment and vascular leak. Nat. Med..

[9] Kaffe E., Katsifa A., Xylourgidis N., Ninou I., Zannikou M., Harokopos V., Foka P., Dimitriadis A., Evangelou K., Moulas A. (2017). Hepatocyte autotaxin expression promotes liver fibrosis and cancer. Hepatology.

[10] Benesch M., Tang X., Venkatraman G., Bekele R., Brindley D. (2016). Recent advances in targeting the autotaxin-lysophosphatidate-lipid phosphate phosphatase axis in vivo. J. Biomed. Res..

[11] Kremer A.E., Martens J.J., Kulik W., Ruëff F., Kuiper E.M., van Buuren H.R., van Erpecum K.J., Kondrackiene J., Prieto J., Rust C. (2010). Lysophosphatidic Acid Is a Potential Mediator of Cholestatic Pruritus. Gastroenterology.

[12] D’Souza K., Paramel G.V., Kienesberger P.C. (2018). Lysophosphatidic Acid Signaling in Obesity and Insulin Resistance. Nutrients.

[13] Barbayianni E., Kaffe E., Aidinis V., Kokotos G. (2015). Autotaxin, a secreted lysophospholipase D, as a promising therapeutic target in chronic inflammation and cancer. Prog. Lipid Res..

[14] Albers H.M.H.G., Ovaa H. (2012). Chemical Evolution of Autotaxin Inhibitors. Chem. Rev..

[15] Houben A.J.S., van Wijk X.M.R., van Meeteren L.A., van Zeijl L., van de Westerlo E.M.A., Hausmann J., Fish A., Perrakis A., van Kuppev T.H. (2013). The polybasic insertion in autotaxin α confers specific binding to heparin and cell surface heparan sulfate proteoglycans. J. Biol. Chem..

[16] Perrakis A., Moolenaar W.H. (2014). Autotaxin: Structure-function and signaling. J. Lipid Res..

[17] Jansen S., Stefan C., Creemers J.W.M., Waelkens E., Van Eynde A., Stalmans W., Bollen M. (2005). Proteolytic maturation and activation of autotaxin (NPP2), a secreted metastasis-enhancing lysophospholipase D. J. Cell Sci..

[18] Leblanc R., Lee S.-C., David M., Bordet J.-C., Norman D.D., Patil R., Miller D., Sahay D., Ribeiro J., Clézardin P. (2014). Interaction of platelet-derived autotaxin with tumor integrin αVβ3 controls metastasis of breast cancer cells to bone. Blood.

[19] Wu T., Kooi C.V., Shah P., Charnigo R., Huang C., Smyth S.S., Morris A.J. (2014). Integrin-mediated cell surface recruitment of autotaxin promotes persistent directional cell migration. FASEB J..

[20] Hashimoto T., Okudaira S., Igarashi K., Hama K., Yatomi Y., Aoki J. (2011). Identification and biochemical characterization of a novel autotaxin isoform, ATX, with a four-amino acid deletion. J. Biochem..

[21] Hausmann J., Kamtekar S., Christodoulou E., Day J.E., Wu T., Fulkerson Z., Albers H.M.H.G., van Meeteren L., Houben A.J.S., Van Zeijl L. (2011). Structural basis of substrate discrimination and integrin binding by autotaxin. Nat. Struct. Mol. Biol..

[22] Nishimasu H., Okudaira S., Hama K., Mihara E., Dohmae N., Inoue A., Ishitani R., Takagi J., Aoki J., Nureki O. (2011). Crystal structure of autotaxin and insight into GPCR activation by lipid mediators. Nat. Struct. Mol. Biol..

[23] Keune W.-J., Hausmann J., Bolier R., Tolenaars D., Kremer A., Heidebrecht T., Joosten R.P., Sunkara M., Morris A.J., Matas-Rico E. (2016). Steroid binding to Autotaxin links bile salts and lysophosphatidic acid signalling. Nat. Commun..

[24] Salgado-Polo F., Fish A., Matsoukas M.-T., Heidebrecht T., Keune W.-J., Perrakis A. (2018). Lysophosphatidic acid produced by autotaxin acts as an allosteric modulator of its catalytic efficiency. J. Biol. Chem..

[25] Jethwa S.A., Leah E.J., Zhang Q., Bright N.A., Oxley D., Bootman M., Rudge S.A., Wakelam M.J.O. (2016). Exosomes bind autotaxin and act as a physiological delivery mechanism to stimulate LPA receptor signalling in cells. J. Cell Sci..

[26] Chen J., Li H., Xu W., Guo X. (2021). Evaluation of serum ATX and LPA as potential diagnostic biomarkers in patients with pancreatic cancer. BMC Gastroenterol..

[27] Nojiri T., Kurano M., Araki O., Nakawatari K., Nishikawa M., Shimamoto S., Igarashi K., Kano K., Aoki J., Kihara S. (2019). Serum autotaxin levels are associated with Graves’ disease. Endocr. J..

[28] Igarashi N., Honjo M., Asaoka R., Kurano M., Yatomi Y., Igarashi K., Miyata K., Kaburaki T., Aihara M. (2021). Aqueous autotaxin and TGF-βs are promising diagnostic biomarkers for distinguishing open-angle glaucoma subtypes. Sci. Rep..

[29] Nie C., Zhang L., Chen X., Li Y., Ha F., Liu H., Han T. (2020). Autotaxin: An Early Warning Biomarker for Acute-on-chronic Liver Failure. J. Clin. Transl. Hepatol..

[30] Bourgeois R., Devillers R., Perrot N., Després A.-A., Boulanger M.-C., Mitchell P.L., Guertin J., Couture P., Boffa M.B., Scipione C.A. (2020). Interaction of Autotaxin with Lipoprotein(a) in Patients with Calcific Aortic Valve Stenosis. JACC Basic Transl. Sci..

[31] Rahman M.A., Tan M.L., Johnson S.P., Hollows R.J., Chai W.L., Mansell J.P., Yap L.F., Paterson I.C. (2020). Deregulation of lysophosphatidic acid metabolism in oral cancer promotes cell migration via the up-regulation of COX-2. PeerJ.

[32] Brindley D.N., Tang X., Meng G., Benesch M.G.K. (2020). Role of Adipose Tissue-Derived Autotaxin, Lysophosphatidate Signaling, and Inflammation in the Progression and Treatment of Breast Cancer. Int. J. Mol. Sci..

[33] Jinno N., Yoshida M., Hayashi K., Naitoh I., Hori Y., Natsume M., Kato A., Kachi K., Asano G., Atsuta N. (2021). Autotaxin in ascites promotes peritoneal dissemination in pancreatic cancer. Cancer Sci..

[34] Zhao Y., Hasse S., Zhao C., Bourgoin S.G. (2019). Targeting the autotaxin-Lysophosphatidic acid receptor axis in cardiovascular diseases. Biochem. Pharmacol..

[35] Sakai N., Bain G., Furuichi K., Iwata Y., Nakamura M., Hara A., Kitajima S., Sagara A., Miyake T., Toyama T. (2019). The involvement of autotaxin in renal interstitial fibrosis through regulation of fibroblast functions and induction of vascular leakage. Sci. Rep..

[36] Kaffe E., Magkrioti C., Aidinis V. (2019). Deregulated Lysophosphatidic Acid Metabolism and Signaling in Liver Cancer. Cancers.

[37] Ninou I., Sevastou I., Magkrioti C., Kaffe E., Stamatakis G., Thivaios S., Panayotou G., Aoki J., Kollias G., Aidinis V. (2020). Genetic deletion of Autotaxin from CD11b+ cells decreases the severity of experimental autoimmune encephalomyelitis. PLoS ONE.

[38] Herr D.R., Chew W.S., Satish R.L., Ong W.-Y. (2020). Pleotropic Roles of Autotaxin in the Nervous System Present Opportunities for the Development of Novel Therapeutics for Neurological Diseases. Mol. Neurobiol..

[39] Fukui M., Tsutsumi T., Yamamoto-Mikami A., Morito K., Takahashi N., Tanaka T., Iwasa T., Kuwahara A., Irahara M., Tokumura A. (2020). Distinct contributions of two choline-producing enzymatic activities to lysophosphatidic acid production in human amniotic fluid from pregnant women in the second trimester and after parturition. Prostaglandins Other Lipid Mediat..

[40] Van Meeteren L.A., Ruurs P., Stortelers C., Bouwman P., van Rooijen M.A., Pradère J.P., Pettit T.R., Wakelam M.J.O., Saulnier-Blache J.S., Mummery C.L. (2006). Autotaxin, a Secreted Lysophospholipase D, Is Essential for Blood Vessel Formation during Development. Mol. Cell. Biol..

[41] Magkrioti C., Galaris A., Kanellopoulou P., Stylianaki E.-A., Kaffe E., Aidinis V. (2019). Autotaxin and chronic inflammatory diseases. J. Autoimmun..

[42] Katsifa A., Kaffe E., Nikolaidou-Katsaridou N., Economides A., Newbigging S., McKerlie C., Aidinis V. (2015). The Bulk of Autotaxin Activity Is Dispensable for Adult Mouse Life. PLoS ONE.

[43] Galperin M.Y., Jedrzejas M.J. (2001). Conserved core structure and active site residues in alkaline phosphatase superfamily enzymes. Proteins Struct. Funct. Bioinform..

[44] Gijsbers R., Ceulemans H., Stalmans W., Bollen M. (2001). Structural and Catalytic Similarities between Nucleotide Pyrophosphatases/Phosphodiesterases and Alkaline Phosphatases. J. Biol. Chem..

[45] Clair T., Aoki J., Koh E., Bandle R.W., Nam S.W., Ptaszynska M.M., Mills G.B., Schiffmann E., Liotta L.A., Stracke M.L. (2003). Autotaxin hydrolyzes sphingosylphosphorylcholine to produce the regulator of migration, sphingosine-1-phosphate. Cancer Res..

[46] Clair T., Koh E., Ptaszynska M., Bandle R.W., Liotta L.A., Schiffmann E., Stracke M.L. (2005). L-histidine inhibits production of lysophosphatidic acid by the tumor-associated cytokine, autotaxin. Lipids Health Dis..

[47] Virag T., Elrod D.B., Liliom K., Sardar V.M., Parrill A.L., Yokoyama K., Durgam G., Deng W., Miller D.D., Tigyi G. (2003). Fatty Alcohol Phosphates are Subtype-Selective Agonists and Antagonists of Lysophosphatidic Acid Receptors. Mol. Pharmacol..

[48] Durgam G.G., Virag T., Walker M.D., Tsukahara R., Yasuda S., Liliom K., van Meeteren L.A., Moolenaar W.H., Wilke N., Siess W. (2005). Synthesis, Structure−Activity Relationships, and Biological Evaluation of Fatty Alcohol Phosphates as Lysophosphatidic Acid Receptor Ligands, Activators of PPARγ, and Inhibitors of Autotaxin. J. Med. Chem..

[49] Durgam G.G., Tsukahara R., Makarova N., Walker M.D., Fujiwara Y., Pigg K.R., Baker D.L., Sardar V.M., Parrill A.L., Tigyi G. (2006). Synthesis and pharmacological evaluation of second-generation phosphatidic acid derivatives as lysophosphatidic acid receptor ligands. Bioorg. Med. Chem. Lett..

[50] Gududuru V., Zeng K., Tsukahara R., Makarova N., Fujiwara Y., Pigg K.R., Baker D.L., Tigyi G., Miller D.D. (2006). Identification of Darmstoff analogs as selective agonists and antagonists of lysophosphatidic acid receptors. Bioorg. Med. Chem. Lett..

[51] Gupte R., Siddam A., Lu Y., Li W., Fujiwara Y., Panupinthu N., Pham T.-C., Baker D.L., Parrill A.L., Gotoh M. (2010). Synthesis and pharmacological evaluation of the stereoisomers of 3-carba cyclic-phosphatidic acid. Bioorg. Med. Chem. Lett..

[52] Altman M.K., Gopal V., Jia W., Yu S., Hall H., Mills G.B., McGinnis A.C., Bartlett M.G., Jiang G., Madan D. (2010). Targeting melanoma growth and viability reveals dualistic functionality of the phosphonothionate analogue of carba cyclic phosphatidic acid. Mol. Cancer.

[53] Nikitopoulou I., Kaffe E., Sevastou I., Sirioti I., Samiotaki M., Madan D., Prestwich G.D., Aidinis V. (2013). A Metabolically-Stabilized Phosphonate Analog of Lysophosphatidic Acid Attenuates Collagen-Induced Arthritis. PLoS ONE.

[54] van Meeteren L.A., Brinkmann V., Saulnier-Blache J.S., Lynch K.R., Moolenaar W.H. (2008). Anticancer activity of FTY720: Phosphorylated FTY720 inhibits autotaxin, a metastasis-enhancing and angiogenic lysophospholipase D. Cancer Lett..

[55] Fisher N., Hilton-Bolt T., Edwards M.G., Haxton K.J., McKenzie M., Allin S.M., Richardson A. (2014). Dendrimer Conjugate of [4-(Tetradecanoylamino)benzyl]phosphonic Acid (S32826) as an Autotaxin Inhibitor. ACS Med. Chem. Lett..

[56] Jiang G., Madan D., Prestwich G.D. (2011). Aromatic phosphonates inhibit the lysophospholipase D activity of autotaxin. Bioorg. Med. Chem. Lett..

[57] Gupte R., Patil R., Liu J., Wang Y., Lee S.C., Fujiwara Y., Fells J., Bolen A.L., Emmons-Thompson K., Yates C.R. (2011). Benzyl and Naphthalene Methylphosphonic Acid Inhibitors of Autotaxin with Anti-invasive and Anti-metastatic Activity. ChemMedChem.

[58] East J.E., Kennedy A.J., Tomsig J.L., De Leon A.R., Lynch K.R., Macdonald T.L. (2010). Synthesis and structure–activity relationships of tyrosine-based inhibitors of autotaxin (ATX). Bioorg. Med. Chem. Lett..

[59] Saunders L.P., Ouellette A., Bandle R., Chang W.C., Zhou H., Misra R.N., De La Cruz E.M., Braddock D.T. (2008). Identification of small-molecule inhibitors of autotaxin that inhibit melanoma cell migration and invasion. Mol. Cancer Ther..

[60] Parrill A.L., Echols U., Nguyen T., Pham T.-C.T., Hoeglund A., Baker D.L. (2008). Virtual screening approaches for the identification of non-lipid autotaxin inhibitors. Bioorg. Med. Chem..

[61] Hoeglund A.B., Bostic H.E., Howard A.L., Wanjala I.W., Best M.D., Baker D.L., Parrill A.L. (2010). Optimization of a Pipemidic Acid Autotaxin Inhibitor. J. Med. Chem..

[62] Albers H.M.H.G., van Meeteren L., Egan D.A., van Tilburg E.W., Moolenaar W.H., Ovaa H. (2010). Discovery and Optimization of Boronic Acid Based Inhibitors of Autotaxin. J. Med. Chem..

[63] Albers H.M.H.G., Dong A., van Meeteren L.A., Egan D.A., Sunkara M., van Tilburg E.W., Schuurman K., van Tellingen O., Morris A.J., Smyth S.S. (2010). Boronic acid-based inhibitor of autotaxin reveals rapid turnover of LPA in the circulation. Proc. Natl. Acad. Sci. USA.

[64] Albers H.M.H.G., Hendrickx L.J.D., van Tol R.J.P., Hausmann J., Perrakis A., Ovaa H. (2011). Structure-Based Design of Novel Boronic Acid-Based Inhibitors of Autotaxin. J. Med. Chem..

[65] Kawaguchi M., Okabe T., Okudaira S., Nishimasu H., Ishitani R., Kojima H., Nureki O., Aoki J., Nagano T. (2013). Screening and X-ray Crystal Structure-based Optimization of Autotaxin (ENPP2) Inhibitors, Using a Newly Developed Fluorescence Probe. ACS Chem. Biol..

[66] Kraljić K., Jelić D., Žiher D., Cvrtila A., Dragojević S., Sinković V., Mesić M. (2019). Benzoxaboroles—Novel Autotaxin Inhibitors. Molecules.

[67] Iwaki Y., Ohhata A., Nakatani S., Hisaichi K., Okabe Y., Hiramatsu A., Watanabe T., Yamamoto S., Nishiyama T., Kobayashi J. (2020). ONO-8430506: A Novel Autotaxin Inhibitor That Enhances the Antitumor Effect of Paclitaxel in a Breast Cancer Model. ACS Med. Chem. Lett..

[68] Nikolaou A., Ninou I., Kokotou M.G., Kaffe E., Afantitis A., Aidinis V., Kokotos G. (2018). Hydroxamic Acids Constitute a Novel Class of Autotaxin Inhibitors that Exhibit in Vivo Efficacy in a Pulmonary Fibrosis Model. J. Med. Chem..

[69] Li X., Xie M., Lu C., Mao J., Cao Y., Yang Y., Wei Y., Liu X., Cao S., Song Y. (2020). Design and synthesis of Leukotriene A4 hydrolase inhibitors to alleviate idiopathic pulmonary fibrosis and acute lung injury. Eur. J. Med. Chem..

[70] Kuttruff C.A., Ferrara M., Bretschneider T., Hoerer S., Handschuh S., Nosse B., Romig H., Nicklin P., Roth G.J. (2017). Discovery of BI-2545: A Novel Autotaxin Inhibitor That Significantly Reduces LPA Levels in Vivo. ACS Med. Chem. Lett..

[71] Jones S.B., Pfeifer L.A., Bleisch T.J., Beauchamp T.J., Durbin J.D., Klimkowski V.J., Hughes N.E., Rito C.J., Dao Y., Gruber J.M. (2016). Novel Autotaxin Inhibitors for the Treatment of Osteoarthritis Pain: Lead Optimization via Structure-Based Drug Design. ACS Med. Chem. Lett..

[72] Gerokonstantis D.T., Nikolaou A., Magkrioti C., Afantitis A., Aidinis V., Kokotos G., Moutevelis-Minakakis P. (2020). Synthesis of novel 2-pyrrolidinone and pyrrolidine derivatives and study of their inhibitory activity against autotaxin enzyme. Bioorg. Med. Chem..

[73] Balupuri A., Lee M.H., Chae S., Jung E., Yoon W., Kim Y., Son S.J., Ryu J., Kang D.-H., Yang Y.-J. (2018). Discovery and optimization of ATX inhibitors via modeling, synthesis and biological evaluation. Eur. J. Med. Chem..

[74] Gierse J., Thorarensen A., Beltey K., Bradshaw-Pierce E., Cortes-Burgos L., Hall T., Johnston A., Murphy M., Nemirovskiy O., Ogawa S. (2010). A Novel Autotaxin Inhibitor Reduces Lysophosphatidic Acid Levels in Plasma and the Site of Inflammation. J. Pharmacol. Exp. Ther..

[75] Rancoule C., Dusaulcy R., Tréguer K., Grès S., Attané C., Saulnier-Blache J.S. (2014). Involvement of autotaxin/lysophosphatidic acid signaling in obesity and impaired glucose homeostasis. Biochimie.

[76] Balupuri A., Lee D.-Y., Lee M.H., Chae S., Jung E., Kim Y., Ryu J., Kang N.S. (2017). Design, synthesis, docking and biological evaluation of 4-phenyl-thiazole derivatives as autotaxin (ATX) inhibitors. Bioorg. Med. Chem. Lett..

[77] Fells J.I., Lee S.C., Fujiwara Y., Norman D.D., Lim K.G., Tsukahara R., Liu J., Patil R., Miller D.D., Kirby R.J. (2013). Hits of a High-Throughput Screen Identify the Hydrophobic Pocket of Autotaxin/Lysophospholipase D As an Inhibitory Surface. Mol. Pharmacol..

[78] Fells J.I., Lee S.C., Norman D.D., Tsukahara R., Kirby J.R., Nelson S., Seibel W., Papoian R., Patil R., Miller D.D. (2014). Targeting the hydrophobic pocket of autotaxin with virtual screening of inhibitors identifies a common aromatic sulfonamide structural motif. FEBS J..

[79] Banerjee S., Norman D.D., Lee S.C., Parrill A.L., Pham T.C.T., Baker D.L., Tigyi G.J., Miller D.D. (2017). Highly Potent Non-Carboxylic Acid Autotaxin Inhibitors Reduce Melanoma Metastasis and Chemotherapeutic Resistance of Breast Cancer Stem Cells. J. Med. Chem..

[80] Banerjee S., Norman D.D., Deng S., Fakayode S.O., Lee S.C., Parrill A.L., Li W., Miller D.D., Tigyi G.J. (2020). Molecular modelling guided design, synthesis and QSAR analysis of new small molecule non-lipid autotaxin inhibitors. Bioorg. Chem..

[81] Stein A.J., Bain G., Prodanovich P., Santini A.M., Darlington J., Stelzer N.M.P., Sidhu R.S., Schaub J., Goulet L., Lonergan D. (2015). Structural Basis for Inhibition of Human Autotaxin by Four Potent Compounds with Distinct Modes of Binding. Mol. Pharmacol..

[82] Bain G., Shannon K.E., Huang F., Darlington J., Goulet L., Prodanovich P., Ma G.L., Santini A.M., Stein A.J., Lonergan D. (2016). Selective Inhibition of Autotaxin Is Efficacious in Mouse Models of Liver Fibrosis. J. Pharmacol. Exp. Ther..

[83] Miller L., Keune W.-J., Castagna D., Young L.C., Duffy E.L., Potjewyd F., Salgado-Polo F., García P.E., Semaan D., Pritchard J.M. (2017). Structure–Activity Relationships of Small Molecule Autotaxin Inhibitors with a Discrete Binding Mode. J. Med. Chem..

[84] Shah P., Cheasty A., Foxton C., Raynham T., Farooq M., Gutierreza I.F., Lejeunea A., Pritcharda M., Turnbulla A., Panga L. (2016). Discovery of potent inhibitors of the lysophospholipase autotaxin. Bioorg. Med. Chem. Lett..

[85] Pantsar T., Singha P.K., Nevalainen T.J., Koshevoy I., Leppänen J., Poso A., Niskanen J.M., Pasonen-Seppänen S., Savinainen J., Laitinen T. (2017). Design, synthesis, and biological evaluation of 2,4-dihydropyrano[2,3-c]pyrazole derivatives as autotaxin inhibitors. Eur. J. Pharm. Sci..

[86] Joncour A., Desroy N., Housseman C., Bock X., Bienvenu N., Cherel L., Labeguere V., Peixoto C., Annoot D., Lepissier L. (2017). Discovery, Structure–Activity Relationship, and Binding Mode of an Imidazo[1,2-a]pyridine Series of Autotaxin Inhibitors. J. Med. Chem..

[87] Desroy N., Housseman C., Bock X., Joncour A., Bienvenu N., Cherel L., Labeguere V., Rondet E., Peixoto C., Grassot J.-M. (2017). Discovery of 2-[[2-Ethyl-6-[4-[2-(3-hydroxyazetidin-1-yl)-2-oxoethyl]piperazin-1-yl]-8-methylimidazo[1,2-a]pyridin-3-yl]methylamino]-4-(4-fluorophenyl)thiazole-5-carbonitrile (GLPG1690), a First-in-Class Autotaxin Inhibitor Undergoing Clinical Evaluation for the Treatment of Idiopathic Pulmonary Fibrosis. J. Med. Chem..

[88] Keune W.-J., Potjewyd F., Heidebrecht T., Salgado-Polo F., Macdonald S.J.F., Chelvarajan L., Abdel-Latif A., Soman S., Morris A.J., Watson A.J.B. (2017). Rational Design of Autotaxin Inhibitors by Structural Evolution of Endogenous Modulators. J. Med. Chem..

[89] Jing T., Miao X., Jiang F., Guo M., Xing L., Zhang J., Zuo D., Lei H., Zhai X. (2018). Discovery and optimization of tetrahydropyrido[4,3-d]pyrimidine derivatives as novel ATX and EGFR dual inhibitors. Bioorg. Med. Chem..

[90] Jiang N., Zhou Y., Zhu M., Zhang J., Cao M., Lei H., Guo M., Gong P., Su G., Zhai X. (2019). Optimization and evaluation of novel tetrahydropyrido[4,3-d]pyrimidine derivatives as ATX inhibitors for cardiac and hepatic fibrosis. Eur. J. Med. Chem..

[91] Lei H., Guo M., Li X., Jia F., Li C., Yang Y., Cao M., Jiang N., Ma E., Zhai X. (2020). Discovery of Novel Indole-Based Allosteric Highly Potent ATX Inhibitors with Great In Vivo Efficacy in a Mouse Lung Fibrosis Model. J. Med. Chem..

[92] Lei H., Li C., Yang Y., Jia F., Guo M., Zhu M., Jiang N., Zhai X. (2020). Structure guided design of potent indole-based ATX inhibitors bearing hydrazone moiety with tumor suppression effects. Eur. J. Med. Chem..

[93] Jia F., Lei H., Chen Y., Li T., Xing L., Cao Z., Zhai X. (2020). Structure-based linker exploration: Discovery of 1-ethyl-1H-indole analogs as novel ATX inhibitors. Bioorg. Med. Chem..

[94] Kawaguchi M., Okabe T., Okudaira S., Hama K., Kano K., Nishimasu H., Nakagawa H., Ishitani R., Kojima H., Nureki O. (2020). Identification of Potent In Vivo Autotaxin Inhibitors that Bind to Both Hydrophobic Pockets and Channels in the Catalytic Domain. J. Med. Chem..

[95] Ren J.-X., Zhang R.-T., Zhang H. (2020). Identifying Novel ATX Inhibitors via Combinatory Virtual Screening Using Crystallography-Derived Pharmacophore Modelling, Docking Study, and QSAR Analysis. Molecules.

[96] Lee M.H., Lee D.-Y., Balupuri A., Jeong J.-W., Kang N.S. (2019). Pharmacophoric Site Identification and Inhibitor Design for Autotaxin. Molecules.

[97] Thomson C.G., Le Grand D., Dowling M., Beattie D., Elphick L., Faller M., Freeman M., Hardaker E., Head V., Hemmig R. (2021). Development of autotaxin inhibitors: A series of tetrazole cinnamides. Bioorg. Med. Chem. Lett..

